# An Epidemiological Assessment of SARS-CoV-2 in the Sewage System of a Higher Education Institution

**DOI:** 10.5334/aogh.4413

**Published:** 2024-08-07

**Authors:** Carmem Cícera Maria da Silva, Carolina Rangel de Lima Santos, Eliomar Pivante Céleri, David Salles, Julia Miranda Fardin, Kamily Fagundes Pussi, Daniel Claudio de Oliveira Gomes, Vinicius de Oliveira Ribeiro, Leila Cristina Konrad-Moraes, Herintha Coeto Neitzke-Abreu, Valdemar Lacerda Júnior

**Affiliations:** 1 PostGraduate Program in Chemistry, Center for Exact Sciences, Universidade Federal do Espírito Santo (UFES), Vitória, Espírito Santo, Brazil; 2 Infectious Diseases Nucleous, Universidade Federal do Espírito Santo (UFES), Vitória, Espírito Santo, Brazil; 3 Postgraduate Program in Health Sciences, Universidade Federal da Grande Dourados (UFGD), Dourados, Mato Grosso do Sul, Brazil; 4 Graduate Program Environmental and Sanitary Engineering. Universidade Estadual do Mato Grosso do Sul (UEMS), Dourados, Mato Grosso do Sul, Brazil

**Keywords:** COVID-19 pandemic, epidemiological monitoring, wastewater-based epidemiological surveillance

## Abstract

*Background:* The World Health Organization declared the end of the COVID-19 pandemic in May 2023, three years after the adoption of global emergency measures. Monitoring of SARS-CoV-2 in sewage underscores its importance due to its effectiveness and cost-effectiveness, highlighting the need to prioritize research on water resources and sanitation.

*Objectives:* The aim of this study was to conduct an epidemiological assessment of SARS-CoV-2 in the sewage system of a higher education institution located in Vitória Espírito Santo State, Maruípe campus.

*Methods:* Over a period of 66 days, from February 6 to April 12, 2023, 15 samples were collected. Each sample consisted of 1 L, collected in 1 hour, with 250 mL collected every 15 minutes. The samples were characterized by assessing their appearance, and pH was measured using a Horiba U-50 multiparameter probe. The extracted RNA was subjected to RT-qPCR using the Allplex™ 2019-nCovAssay Seegene kit.

*Results:* The samples exhibited a cloudy appearance with impurities, and the pH ranged from 6.35 to 8.17. Among the evaluated samples, SARS-CoV-2 RNA was detected in two, and, by comparing this with the epidemiological bulletin issued by the State Health Department, an increase in cases in the state was observed during the collection period of these samples.

*Conclusions:* Sewage monitoring proved to be an important tool in this post-pandemic period, serving as an alert and prevention mechanism for the population in relation to new outbreaks. Furthermore, it represents a low-cost mapping strategy and extensive testing of a population, aligning with the studies presented at the beginning of the pandemic. We recommend specific adjustments considering distinct populations.

## Introduction

Historically, 2020 will be remembered as the year that changed the routine of people worldwide, due to the pandemic caused by SARS-CoV-2 [1], a virus from the *Coronaviridae* family, common in many animal species and possessing infectious and transmissible potential [2, 3]. During the pandemic peaks, there was a collective experience of fear of death in the most everyday actions. As of January 23, 2024, in Brazil 38,264,864 cases and 708,999 deaths have been recorded [4].

In May 2023, through a communication presented by the World Health Organization (WHO) three years after the adoption of the global emergency, the end of the pandemic was declared. COVID-19 has become an endemic disease, meaning that it still continues to affect people, with some cases resulting in death, as observed in recent epidemiological bulletins released by the Brazilian government health system [5]. In light of the experiences gained during the pandemic, some protocols can be optimized by society, governments, and researchers to monitor and minimize new outbreaks.

The first positive results for the presence of SARS-CoV-2 in sewage samples were published in the Netherlands when investigating the presence of viral RNA by RT-qPCR in wastewater from seven sewage treatment plants and an airport [6, 7]. These studies highlight the epidemiological monitoring of SARS-CoV-2 in sewage as a tool with great potential due to its cost-effectiveness and robustness, emphasizing that studies on the role of water resources and sanitation should be prioritized, given the potential to serve as a health indicator. A study by Wu et al. [8] demonstrated that in symptomatic and asymptomatic patients, feces contained viral load for approximately 30 days.

During the pandemic, routine testing of the entire population would be an excellent preventive measure, but one of great complexity, especially in populous and third-world countries, due to high costs and the complexity of conducting tests, especially with children. Epidemiological study patterns for effluents are already implemented in monitoring hepatitis A virus, polio, norovirus, and rotavirus [9]. Therefore, the aim of this study was to evaluate an epidemiological study of SARS-CoV-2 in the sewage system of a higher education institution, with the intention of emphasizing this procedure as a strategic standard for mapping SARS-CoV-2 in the post-pandemic period.

## Materials and Methods

A technical visit for the assessment and planning of sewage system sampling was conducted at the Universidade Federal do Espírito Santo (UFES), located in the Maruípe neighborhood, in Vitória, the capital of Espírito Santo. The Maruípe campus houses the Health Sciences Center (CCS)—with undergraduate courses in medicine, dentistry, pharmacy, nursing, physiotherapy, nutrition, speech therapy, and occupational therapy—as well as the Cassiano Antonio Moraes University Hospital (HUCAM).

**Figure 1:**
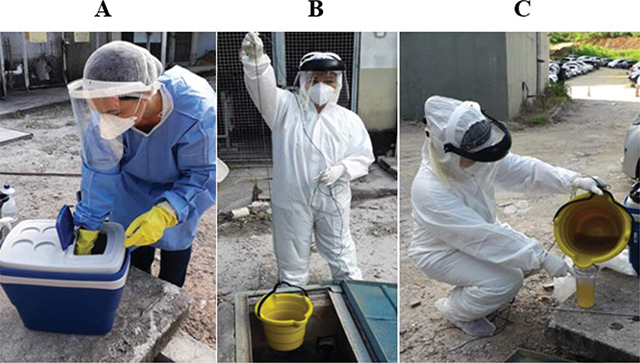
Execution of the sample collection procedures.

Samples were collected during the peak sewage flow, aiming for the highest representativity of the population, at two collection points (UFES-CCS and HUCAM) that gathered all the sewage from their respective units. Fifteen samples were collected over a period of 66 days, from February 6 to April 12, 2023. Each sample consisted of 1 L, collected in 1 hour, with 250 mL collected every 15 minutes. Sterile glass containers were used and stored in a thermal box at 10°C (Figure 1A). The collection was carried out by adapting ropes to buckets due to the specificity of the location (Figure 1B). For each collection, a new bucket-and-rope system was used to mitigate contaminations. Pre-filtrations were performed using stainless steel sieves during the collections (Figure 1C). All biosafety procedures, such as the use of individual and collective protective equipment, were employed (Figure 1C). After the completion of the collections, the samples were transferred to the Biosafety Level 2 Laboratory at the UFES Infectious Diseases Nucleous.

## Evaluation of Appearance and pH Verification

**Figure 2:**
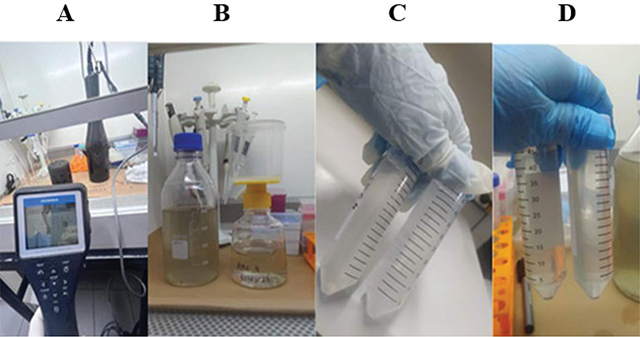
pH determination and concentration of the sewage sample.

In the samples collected from raw sewage, a visual assessment of the appearance and pH verification was conducted using the Horiba U-50 multiparameter probe (Figure 2A).

## Sample Processing

Initially, the sample was homogenized, and centrifugation was performed at 4°C for 5 minutes at 1800 g. The supernatant was filtered through a sterile vacuum filtration system with a PES (Polyethersulfone) 0.22 µm membrane (Figure 2B). After filtration, 40 mL of the filtrate was transferred to a tube, and 4.0 g of Polyethyleneglycol Bio Ultra 8000 (Sigma, Darmstadt, Germany) and 0.9 g of NaCl were added, shaken for 15 minutes for complete dissolution (Figure 2C). Subsequently, centrifugation was carried out for 60 minutes at 4°C at 1800 g. After centrifugation and pellet formation (Figure 2D), the supernatant was carefully removed, the pellet eluted in 1% PBS (sodium phosphate 10 mM; NaCl 0.15 M, pH 7.2), transferred to a cryotube, stored at −80°C, and submitted to the Research Laboratory in Health Sciences at the Universidade Federal da Grande Dourados.

## Molecular Analysis of SARS-CoV-2

The viral RNA from the samples was extracted using the Quick-DNA/RNA Viral MagBead Kit (Zymo Research, CA, US). Subsequently, the samples were stored at −80°C until analysis. The extracted RNA underwent RT-qPCR using the Allplex™ 2019-nCovAssay Seegene kit, following the manufacturer’s instructions, on the BioRad CFX 96 Touch Real-Time thermocycler. Viral concentrations were determined by the Ct (threshold cycle) values from the RT-qPCR assays, with specific primers for the Sarbecovirus E gene in the FAM channel, COVID-19 RdRP gene in the Cal Red 610 channel, COVID-19 N gene in the Quasar 670 channel, and human internal control in the HEX channel. The kit used (Allplex™ 2019-nCovAssay) has a detection limit of 4167 copies/mL (100 RNA copies/reaction) (Seracare, Accuplex™ SARS-CoV-2).

## Sequencing of SARS-CoV-2

The SARS-CoV-2 positive samples were sent for sequencing to the Aggeu Magalhães Institute (Fiocruz-Pernambuco). Single-strand cDNA was generated using the High-Capacity cDNA Reverse Transcription kit (Thermo Fisher Scientific Baltics UAB V.A. Graiciuno 8, LT-02241 Vilnius, Lithuania) following the manufacturer’s recommendations. Subsequently, amplicon tailing was performed with the Arctic 5.2.3 version primer sets to amplify the entire SARS-CoV-2 genome, and library preparation was carried out following the COVID Seq protocol (Illumina Inc.). Sequencing was conducted on a MiSeq Illumina machine using a paired-end approach of 150 base pairs.

For quality control of raw sequencing reads, removal of low-quality reads, genome assembly, and lineage assignment, ViralFlow 1.0.0 (https://viralflow.github.io/) [10] was utilized. This tool performs reference-based genome assembly and runs Pangolin v4.3.1 [11] to assign SARS-CoV-2 lineages. Only genomes with a coverage breadth higher than 70% and assigned lineage were reported.

## Results and Discussion

Wastewater-based epidemiology, described by Foladori et al. [12], involves the use of human markers in protocols aimed at assessing lifestyle, health, and population exposure. Wastewater monitoring enables the early identification of viral segments and, through strategic planning, has the potential to reduce disease spread. Studies indicate that epidemiological monitoring of sewage networks incurs lower costs compared to individual testing of an assessed population, making it essential in developing countries for decision-making, particularly in a pandemic scenario [6]. In this study, the obtained results underscore the importance of sewage network monitoring, as evidenced by comparisons with weekly epidemiological bulletins issued by the Department Health of Espírito Santo State.

## Evaluation of Appearance and pH Verification

**Figure 3:**
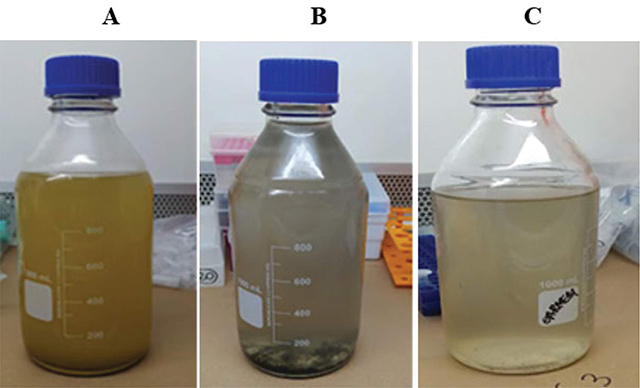
Appearance of the studied sewage samples.

The collected samples appeared turbid (Figure 3A–C), with a substantial amount of suspended solid materials such as fecal residues, despite pre-filtration being conducted in the field. In some samples, the formation of a dark solid at the bottom of the container was observed. The pH ranged from 6.35 to 8.17, with no abrupt variation from neutral pH.

According to Michalake, Silva, and Silva [13], sewage in Brazil is often inadequately treated, and it is frequently discharged into receiving bodies. The pH values for sewage in the treatment process can vary between 5.0 and 9.0. In their evaluation of sewage from influents and effluents at a sewage treatment plant in Curitiba [13], they obtained a pH range of 6.9 to 7.2. A study conducted in the South of Minas Gerais did not observe significant relationships between pH and detected viral load [14], despite scientific literature indicating that the concentration of SARS-CoV-2 can be influenced by the physical and chemical characteristics of sewage [15]. In the present study, no direct relationship with pH was observed among samples positive for SARS-CoV-2 RNA.

## Molecular Analysis

In our study, SARS-CoV-2 RNA was detected in two samples collected on February 7, 2023 (HUCAM) and April 12, 2023 (CCS), and the Ct values found were as follows: for the E gene (32.46 and 32.55), RdRP gene (37.03 and 35.42), and N gene (32.83 and 32.05), respectively (Table 1), suggesting the presence of viral RNA but with a low viral load [16, 17]. It was not possible to identify the SARS-CoV-2 lineage through sequencing as the samples had a low viral load (Ct below 35).

**Table 1:** Sewage samples collected between February and April 2023 from Cassiano Antonio Moraes University Hospital (HUCAM) and Health Sciences Center (CCS), Brazil.

SAMPLE	COLLECTION DATE	COLLECTION SITE	CYCLE THRESHOLD (CT) OF GENES		
			E	RDRP	N
S1	February 6	HUCAM	>40	>40	>40
S2*	February 7	HUCAM	32.46	37.03	32.83
S3	March 10	CCS	>40	>40	>40
S4	March 27	CCS	>40	>40	>40
S5	March 27	CCS	>40	>40	>40
S6	March 28	CCS	>40	>40	>40
S7	March 28	CCS	>40	>40	>40
S8	March 29	CCS	>40	>40	>40
S9	March 29	CCS	>40	>40	>40
S10	April 4	CCS	>40	>40	>40
S11	April 4	CCS	>40	>40	>40
S12	April 6	CCS	>40	>40	>40
S13	April 6	CCS	>40	>40	>40
S14	April 11	CCS	>40	>40	>40
S15*	April 12	CCS	32.55	35.42	32.05

* Positive samples.

The State Health Department of Espírito Santo issued weekly epidemiological bulletins to analyze and disseminate information about COVID-19 cases and their transmission in the state. During the investigated period, the bulletins indicated that positive samples were found between epidemiological weeks 6 (EW-6) and 15 (EW-15) [18]. The city of Vitória, the capital of the state, is divided into 2 districts called Vitória and Goiabeiras, subdivided into nine Administrative Regions (RAs) and 80 neighborhoods [19]. The study took place on the Maruípe campus, located in the Vitória district, in the fourth RA, covering an area of 5,684,216 m² with a population of 54,402. During the collection period, 1382 cases were reported in Vitória, with a significant increase from EW-6 (42 cases) to EW-15 (215 cases). This demonstrates the presence of individuals infected with SARS-CoV-2 during that period [18].

At the state level, the State of Espírito Santo has an overall cumulative value of 1,354,059 COVID-19 cases [18]. Nationally, the overall cumulative number in April 2023 was 37,287,971 million cases of COVID-19, with 753,453 new cases reported from February to April 2023 in Brazil [5].

In Brazil, some institutions have detected SARS-CoV-2 RNA in sewage samples, both treated and untreated [20, 21]. Several studies have also shown the presence of SARS-CoV-2 RNA in sewage, often preceding viral circulation in the population [22–26].

Different groups are also monitoring and detecting SARS-CoV-2 in sewage samples [27–30]. Consequently, a COVID-19 Wastewater Monitoring Network was established in 2020 in Brazil, through collaboration between the National Water Agency, the Ministry of Science and Technology and Innovations, and the Ministry of Health, to develop an early warning system for COVID-19 [31]. The Network issued weekly epidemiological monitoring reports in various regions of the country, including São Paulo, Foz do Iguaçu (Paraná State), Goiânia (Goiás State), and the Distrito Federal, indicating that an increase in viral concentration in sewage likely corresponds to an increase in the studied population and vice versa [6].

## Conclusions

Though the WHO declared that the pandemic ended, it is understood that the end of the pandemic does not signify the end of COVID-19, but rather that precautions and care need to be maintained. In this study, it was observed through epidemiological assessment of sewage that SARS-CoV-2 is still circulating within a university population. Individuals in this population were actively engaged in work or study activities and may or may not have been symptomatic. Detection of the virus in sewage can provide valuable information about the presence and spread of SARS-CoV-2 within a community, contributing to public health decision-making and endemic control. Therefore, the benefits of implementing sewage sampling for SARS-CoV-2 detection generally outweigh the costs involved. Although the protocol was not adopted for surveillance purposes, as the pandemic ended with widespread vaccination, this assessment underscores the importance of sewage monitoring as an essential tool in the post-pandemic period, serving as an alert and prevention measure for potential new outbreaks and epidemics among the population. The cost is considered low compared to individual human testing of a specific population. Recognizing that populations are diverse in terms of cultural, socioeconomic, geographical, and other characteristics, it is recommended that surveillance and monitoring policies for SARS-CoV-2 in wastewater need to be adapted considering specific epidemiological differences or patterns within certain communities.

## Data Availability

All data set is within the manuscript.
